# Improving Hepatitis B Vaccine Efficacy in End-Stage Renal Diseases Patients and Role of Adjuvants

**DOI:** 10.5402/2012/960413

**Published:** 2012-09-18

**Authors:** Mohammad Hossein Somi, Babak Hajipour

**Affiliations:** ^1^Liver and Gastroenterology Research Center, Tabriz University of Medical Sciences, Tabriz, Iran; ^2^Urmia University of Medical Sciences, Urmia, Iran

## Abstract

Hepatitis B virus (HBV) infection is a serious global health problem.The prevalence of viral hepatitis is higher in dialysis patients than in the general population because of the opportunity for exposure during the dialysis procedure. Immunization is the most effective way to prevent transmission of hepatitis B virus (HBV) and hence the development of acute or chronic hepatitis B. It is well established that patients with end-stage renal disease including dialysis-dependent patients, have an impaired immune response to hepatitis B vaccine. End stage renal diseases (ESRD) patients have lower seroconversion rates compared with the subjects with intact renal function. Moreover, even after the completion of vaccination schedule anti-hepatitis B (anti-HBs) titers of responder dialysis, patients are low and decline logarithmically with time. The impaired efficacy of HBV vaccine in patients with ESRD has been attributed to numerous factors such as immune compromise because of uremia and some other factors. One approach to improve the immunogenicity of existing HBV vaccines is adjuvantation, and it's very important to find more effective adjutants for improving HBV vaccine efficacy. In this paper we have a brief review on recently known new ways for improving HBV vaccine efficacy.

## 1. Hepatitis B Virus (HBV)

Hepatitis B virus (HBV) is an important cause of serious liver disease including acute and chronic hepatitis, cirrhosis, and primary hepatocellular carcinoma. Liver disease is a significant cause of morbidity and mortality in patients on maintenance dialysis, with hepatitis HBV infection among the important etiologies. People under going chronic dialysis are at high risk for HBV infection. People with chronic HBV infection can transmit the infection for many years. There are probably more than 400 million people worldwide with chronic hepatitis B infection which is responsible for 520,000 deaths and 470,000 cases of hepatocellular carcinoma (HCC) annually [1]. More than 75% of these live in the Asia-Pacific region, with high numbers also residing in Africa and the Amazon basin. In areas of high endemicity, the lifetime infection rate is above 50%, and more than 8% of the populations are chronic carriers [2]. Infection in such regions is typically acquired in childhood, either horizontally from other children or perinatally from maternal carriers. By contrast, parenteral transmission is common in Australia, and fewer than 2% of the populations are chronic HBV carriers [3].

## 2. Hepatitis B and Hemodialysis Patients

The prevalence of HBV infection is now low within dialysis units of the developed world with chronic HBsAg seropositivity ranging from 0% to 10% in patients on long-term dialysis. Recently, a study sample from the dialysis outcome and practice patterns study, a cross-sectional study of 8615 adult haemodialysis patients from 308 dialysis facilities in Western Europe and the United States, has reported that the prevalence rates of chronic HBV infection on regular dialysis range between 0% and 7% [4]. The majority of facilities (78.1%) had an HBsAg seroconversion rate of 0 per 100 patient-years [4].

The epidemiology of HBV among dialysis patients in the less-developed world is not well known. There are scattered reports, typically single-center surveys, with rates of chronic HBsAg carriers ranging between 2% and 20% [5–13]. The higher HBV infection rates within dialysis units in the developing world can be attributed to several factors, an important one being the higher background prevalence of HBV in the general population. Also playing a prominent role are difficulties following infection control strategies against HBV such as “standard” precautions, routine hemodialysis precautions, cohorting of HBsAg-positive patients by rooms, machines and staff, vaccination against HB virus, and blood screening; these deficiencies are often attributable, at least in part, to a lack of financial and other resources [14, 15]. 

Over the past few decades, there has been a substantial decrease in the incidence of hepatitis B virus (HBV) infection in hemodialysis patients, probably attributable to screening of blood donors, a decline in blood transfusion requirements with increased erythropoietin use, and authoritative guidelines relating to infection control and vaccination. Despite this progress, hemodialysis patients remain at increased risk of acquiring HBV because of increased exposure to blood products, shared hemodialysis equipment, frequent breaching of skin, immunodeficiency, and continuing high prevalence rates of HBV infection among hemodialysis populations.

It is well established that patients with end-stage renal disease (ESRD), whether dialysis dependent or not, have an impaired immune response to hepatitis B vaccine [15]. ESRD patients have lower seroconversion rates compared with the subjects with intact renal function. Moreover, even after the completion of vaccination schedule anti-hepatitis B (anti-HBs) titres of responder dialysis, patients are low, and decline logarithmically with time [15]. Recent reports [16, 17] have demonstrated a low but not inconsequential frequency of hepatitis B virus (HBV) infection in dialysis facilities in the developed countries. Prevalence and incidence rates of HBV are higher within the dialysis units of the developing world [18–21]. Outbreaks of HBV infection among the haemodialysis (HD) patients continue to be recognized in the industrialized countries [22]. The control of spread of HBV infection within dialysis units of developed world has been a milestone in the management of ESRD [15]. The impaired efficacy of HBV vaccine in patients with ESRD has been attributed to numerous factors such as immune compromise because of uraemia, age [23], gender [24], body weight [25], nutritional status [26], seropositivity for antibody against hepatitis C virus (HCV) [27] or human immunodeficiency virus (HIV) [28], low serum albumin [29], possession of the major histocompatibility complex haplotype HLA-B8, SCOI, DR3 [30], and blood transfusion history [31]. The failure to complete a full course of HBV vaccination also impairs the immunization response [32, 33]. Although acute infection tends to be mild and asymptomatic in dialysis patients, up to two-thirds may progress to chronic carriage, with significant risk of chronic liver disease, premature death from cirrhosis or liver cancer, and nosocomial transmission within hemodialysis units [34–36].

## 3. Epidemiology of HDV among Hemodialysis Patients

Twenty-five years ago Rizzetto et al. [37], while examining liver biopsies from individuals infected with HBV, discovered by immunofluorescence a previously unrecognized nuclear antigen that was subsequently shown to be a specific marker of a novel human pathogen, HDV. 

The clinical association with HBV results from the fact that HDV is a defective virus that requires a helper function provided by HBV or other hepadnaviruses [38]. In association with HBV, HDV produces significantly more severe illness than HBV alone [39]. HDV is now well known to induce a spectrum of both acute and chronic liver diseases [40]. Individuals having HBV-HDV coinfection may have more severe acute disease and higher risks of fulminant hepatitis, cirrhosis, and hepatocellular carcinoma (HCC) than those having HBV infection alone [41, 42].

HDV infection has high prevalence in Asia pacific region. Countries like Pakistan and Iran have shown an increase in HDV prevalence over a period of time. Other countries and regions like China, Turkey, Australia, Japan, India, and Taiwan, some of which had very high HDV prevalence in the past, have shown a decline in the incidence, but high prevalence persists in some. Intravenous drug abusers, homosexual men and women, prostitutes, and people on hemodialysis are the groups with very high HDV prevalence [43]. In 2000, 1.3% of blood donors positive for HBsAg and 25.2% of HBsAg-positive hemodialysis patients were found to be anti-HDV positive [44].

In our previous research the seroprevalence of HDV was 9.3% among HBV positive patients. This rate was significantly higher after reaching 40 years of age. The rate was 12.7% in patients with chronic hepatitis B and 4.7% in patients with inactive hepatitis B [45].

## 4. The Hepatitis B Vaccine

In the 1970s, Krugman observed that HBsAg was immunogenic, and that anti-HBs antibodies were protective against hepatitis B [46]. A first-generation vaccine was subsequently developed, consisting of HBsAg extracted by plasmapheresis from HBV carriers, and then inactivated [47]. Safety data are comprehensive. A large prospective trial has shown the vaccine to be safe and well tolerated [48].

Currently available hepatitis B (HB) vaccines have an excellent safety and immunogenicity profile, conferring seroprotection in more than 95% of the vaccinated population [49]. Nevertheless, certain population subgroups, such as some healthy people and immunocompromised subjects, do not respond adequately to vaccination. Among these groups, end-stage renal disease (ESRD) patients, comprising pre- and hemodialysis patients, are considered at high risk for HB infection due to cross-contamination to patients via environmental surfaces, disposables, or equipment during the process of hemodialysis [50–53]. Once infected, about 60% of hemodialysis patients will become chronic carriers of the HB surface antigen (HBsAg), increasing the risk of contamination for other hemodialysis patients, medical personnel, and family members [54] and leading to significant logistic and practical difficulties, including provision for separate medical devices and staff [55]. In a research by Sorkhi et al., patients received 4 microg vaccine intramuscularly at 0, 1, and 6 months. All were negative for HBV infection markers (HBcAb, HBsAg, and HBsAb). Of 62 patients, 53 (85.5%) responded to vaccination, and 26 (49.1%) were high responders [56]. In a research by Miłkowski et al. the efficacy of HBV vaccination was 77.5% in hemodialysis patients [57].

Attempts to overcome the impaired immune response in hemodialysis patients have produced mixed results. An increased dose strategy with additional injections was found to be necessary to improve the response rate in these subjects. Currently a 0-, 1-, 2-, and 6-month schedule with double doses hepatitis B surface antigen (2 × 20 *μ*g HBsAg) of commercially available HB vaccine is recommended in hemodialysis patients, with regular monitoring of antibody levels to ensure that antibody concentrations remain above the protective level of 10 mIU/mL [58]. Several strategies to enhance the immune response have been proposed among which are novel adjuvant systems. Adjuvants are thought to improve immune responses by (a) causing depot formation at the injection site; (b) increasing the interaction between immunogen and macrophage; (c) improving antigen presentation to T cells [59]. Very few vaccine adjuvants have been licensed for prophylaxis in human. Among them alum (aluminum salts) has been widely used for more than 70 years and until recently represented the only adjuvant approved in the United States. Oil in water emulsions (MF59 and AS03) is licensed for adjuvanted influenza vaccines in Europe. AS04, a combination adjuvant composed of monophosphoryl lipid A (MPL) adsorbed to alum, is approved for HBV and human papilloma virus (HPV) vaccines in Europe and has been recently licensed in the USA. This section will focus on the new adjuvants used for improving HBV vaccination efficacy in ESRD patients [60, 61]. 

## 5. Recombinant Interferon-***α***2b

Among the different cytokine candidates containing adjuvant activity, interferons (*α*, *β*, *γ*) deserve special attention in view of their known effects promoting cellular and humoral immune responses [62, 63]. In particular, studies in mouse models have pointed out that interferon (IFN)*α* potently enhanced both T cell and antibody responses, augmenting the production of all subclasses of IgG, and induced long-term antibody production and immunological memory after a single injection of soluble protein antigen [64]. Furthermore, results also obtained in mice have shown that (i) dendritic cells (DCs) are target cells for the adjuvanticity of IFN-*α* *in vivo* [50]; (ii) the administration of this cytokine as an adjuvant of the human influenza vaccine results in a remarkable enhancement of vaccine immunogenicity [65]. Of note, IFN-*α* has been found to strongly induce the differentiation and activation of both mouse and human DCs [66]. Moreover, in chimeric mouse models susceptible to human immunodeficiency virus (HIV) infection, a strong protective immune response has been demonstrated after vaccination with inactivated-virus-pulsed DCs generated from monocytes in the presence of IFN-*α* [67]. Considering all these studies in animal models, we now know that the most effective way of using IFN-*α* as a vaccine adjuvant is when this cytokine is injected together with, or in close proximity to, antigens in order to allow optimal interactions with IFN-primed DCs, thus providing a new rationale for using IFN-*α* as a vaccine adjuvant in humans.

In conclusion, the use of IFN as an immune adjuvant to HBV vaccine is safe and achieves an earlier and higher seroprotection rate improving Th1-dependent immune response in HD patients, suggesting that a similar strategy of IFN-adjuvanted HBV vaccination could be useful in other populations of HBV vaccine hyporesponders, such as cirrhotics and liver transplant recipients, who are at high risk of acquiring HBV infection [68].

## 6. GM-CSF

There is growing evidence that granulocyte macrophage colony-stimulating factor (GM-CSF) enhances the immune response to vaccines direct against both infectious agents and various cancers [69]. GM-CSF has a variety of effects on immune responses, and coimmunization with GM-CSF has been shown to increase the antibody response and to enhance the proliferative response of T cells [70]. The efficacy of GM-CSF as adjuvant to hepatitis B vaccine has been object of several clinical trials conducted in healthy subjects, patients with end-stage renal disease, and HIV-infected patients [71–73].

Early reports have found a very promising effect of GM-CSF as an adjuvant to HBV vaccine [71]. Subsequently, the efficacy of this approach has been widely explored, and a number of controlled and uncontrolled clinical trials have evaluated the adjuvancy properties of GM-CSF in combination with HBV vaccine [73].

The vaccine adjuvant properties of GM-CSF are based on a variety of effects on immune responses, which include macrophage activation, increasing MHC class II antigen expression, enhancing cell maturation and migration, enhancing memory cell generation via T and B cell activation, and inducing localized inflammation at the site of injection [74]. However, the exact mechanism by which GM-CSF may improve the immune response to HBV vaccination is unclear and deserves further research. Of note, a study investigating the effect of GM-CSF in primary nonresponding hemodialysis patients has shown a decrease in the antigen-presenting capacity of peripheral blood mononuclear cells and in the number of circulating dendritic cells [73].

The GM-CSF dosages ranged from 20 to 300 *μ*g, and these differences have been described as a possible explanation for discrepancies of results obtained in different studies. Actually, using logistic regression Cruciani et al. have shown that the treatment effect was dependent on GM-CSF dose. Predicted response rate strongly increased with increase in GM-CSF dosage. Data from studies conducted in hemodialysis patients show that in individuals who have responded to the vaccine GM-CSF increased the anti-HBs titers. In fact, the standardized mean difference of antibody titers at the end of vaccination cycle was statistically significant. Since the extent of the maximal antibody response has been correlated with the persistence of protective antibody over time, it may be supposed that GM-CSF provides a lasting protection in renal failure patients responding to immunization [75, 76].

## 7. HB-AS04

An adjuvanted HBV vaccine HB-AS04 (FENDrix; GlaxoSmithKline (GSK) Biologicals, Rixensart, Belgium) was licensed in Europe in 2005 for active immunization against HBV for patients with renal insufficiency (including prehemodialysis and hemodialysis patients) aged over 15 years [77]. HB-AS04 consists of recombinant hepatitis B surface antigen (HBsAg) formulated with aluminum phosphate and monophosphoryl lipid (MPL), a purified, detoxified derivative of the lipopolysaccharide molecule of the bacterial wall of *Salmonella minnesota* [78]. Results of clinical studies show HB-AS04 to elicit higher and more persistent levels of anti-HBs antibodies than double doses of a conventional recombinant HBV vaccine when administered according to a 0-, 1-, 2-, and 6-month schedule [79].

HB-AS02 is an adjuvanted HBV vaccine containing recombinant HBsAg formulated with AS02 instead of AS04. AS02 is an oil-in-water emulsion-based adjuvant system consisting of MPL and QS21, a highly purified immunostimulant extracted from the bark of the South American Quillaja saponaria tree [77]. HB-AS02 does not contain any aluminum salt and is formulated without any preservative. HB-AS02 administered according to a 0-, 1-, and 10-month schedule has been shown to elicit strong and persistent antibody responses against HBsAg in healthy adult volunteers [80]. All subjects were seroprotected after two vaccine doses, with a geometric mean anti-HBs antibody concentration (GMC) of approximately 8000 mIU/mL [80].

## 8. Comparison of AS02 and AS04

In summary, a three-dose primary course of the adjuvanted HBV vaccine HB-AS02 induces more rapid, enhanced, and persistent protection in pre-dialysis, peritoneal dialysis, and hemodialysis patients than a four-dose primary course of HB-AS04 (FENDrix), an adjuvanted HBV vaccine licensed in Europe for use in patients with renal insufficiency [77]. The higher postvaccination GMCs for anti-HBs antibodies and the greater proportion of subjects achieving anti-HBs antibody concentrations of X100 mIU/mL after vaccination with HB-AS02 suggest that duration of protection is also likely to be enhanced, potentially affording further reductions in the need for booster doses in this at-risk population. Such adjuvanted hepatitis B vaccines may also have clinical utility in other categories of immunocompromised patients [81].

## 9. Erythropoietin (EPO)

Inhibition of erythropoiesis is the main cause of renal anaemia in ESRD patients, and the leading causes of impaired erythropoiesis are inadequate intrinsic EPO production and iron deficiency [82]. With the goal of corrected renal anaemia, recombinant human erythropoietin (rHuEPO) and intravenous (IV) iron replacement therapy are commonly used in ESRD patients. Because of their impaired immune system, patients with renal failure have a suboptimal response to the hepatitis B vaccine [83]. Many authors had tried to search for the factors influencing the antibody response to the hepatitis B vaccine in dialysis patients. There is much evidence suggesting that rHuEPO may influence the immune response because of its effects on the cells of the humoral and cellular immune system [84]. Besides, iron is known to negatively affect cell-mediated immune effector mechanisms directed against invading microorganisms and tumour cells [85]. In conclusion, Liu et al.'s study reveals that rHuEPO treatment improves the hepatitis B vaccination response, and the immune response is positively correlated with the dose of rHuEPO treatment during the vaccinated period in ESRD patients. More importantly, concomitant IV iron therapy will attenuate anti-HBsAg titres after quadruple hepatitis B vaccinations in dialysis patients who are undergoing rHuEPO therapy. In particular, decreased anti-HBsAg titres in the seroconversion group were found [86].

## 10. Levamisole

Levamisole is an immunostimulant that both *in vivo* and *in vitro* has been reported to increase natural killer cells and activated T cells by enhanced production of, or eliciting synergistic effect of, interleukin (IL)-1 [87], IL-2 [88], IL-18, and IL-12 [89]. It has also been reported that levamisole can cause a 3-fold increase in production of interferon *α*/*β* by adherent nonparenchymal liver cells [90]. Studies have reported the effects of levamisole in immunotherapeutic combinations for infectious diseases such as leprosy [91], brucellosis [92], chronic HBV infection [93], and influenza [94]. Other studies have reported on levamisole used as adjuvant therapy for some malignant and preneoplastic tumors [88, 95]. However, few studies have found promising results, especially in the treatment of chronic HBV infection [96, 97].

The results from a meta-analysis by Alavian and Tabatabaei [98] suggested that oral levamisole administered as an adjuvant to HBV vaccine increased seroprotection in these patients with ESRD who were undergoing dialysis. Large randomized clinical trials (RCTs) with longer durations of followup are needed to support this finding as there is a study by Sali et al. [92] levamisole did not increase HB vaccine efficacy significantly.

## 11. Hepatitis B and A Combined Vaccination

Recent reports suggest that combined vaccination of hepatitis B and hepatitis A may improve immunogenicity to hepatitis B in healthy individuals. In 1 study that compared the geometric mean of anti-HBsAg titers at month 6, patients receiving the combined vaccine showed a statistically significant higher response than with monovalent vaccines [99], other studies also reflect the same trend at varying points in the vaccination series [100].

In comparison to healthy persons, who have a reported 89% seroprotection rate at month 3 and 90%–95% at month 7 with standard dosing, seroprotection rates in this study population were much lower [101]. This is not surprising because chronic renal failure often is associated with compromised immune function [102]. However, in our population, the seroprotection rate in our control group also was lower compared with previous studies of hemodialysis patients, in which median seroprotection rates of 64%–86% were reported [103]. There are a number of factors that decrease response to hepatitis B vaccination, including sex, age, HLA type, and nutritional status. 28 of these older age and male sex may have had a significant impact in decreasing seroconversion rates in this study [104].

Vaccination against hepatitis A virus in dialysis patients is not routinely performed because it is neither associated with hemodialysis therapy nor transmitted through parenteral mechanisms [105]. There is also no documented evidence for adverse events associated with vaccination of patients previously immune to hepatitis A virus, although these patients may experience a postvaccination increase in the geometric mean titer of antihepatitis A virus antibody.

In conclusion, there was a statistically significant difference in seroprotection between the 2 groups at the completion of the vaccination series. Vaccination of hemodialysis patients with a combined hepatitis A and hepatitis B regimen may be more effective than hepatitis B monovalent vaccine in providing seroprotection against hepatitis B virus [106]. But as our previous report HAV seroprevalence is high in the society (97.3%) and chronic liver diseases patients (96.5%) in Iran [107], so combined vaccination of HAV and HBV may not be effective, and we recommend that before HAV combined vaccination as adjuvant, it is better to measure HAV antibody, as if it is possible combined vaccination will have no additional effect in stimulating immune system. 

## 12. Dermal or Muscular Route of Administration?

To improve vaccination response, it is advised that patients be vaccinated as early in the course of renal disease as possible, using double-vaccine dose [108] and a 4- rather than 3-dose schedule. Other strategies that have shown variable success include the addition of adjuvants or immunostimulants [109]. Repeated intradermal (ID) administration of low doses of recombinant HBV vaccine has also been tried in small studies [110] with suggested benefit. However, current guidelines 2 advise against ID vaccination, claiming insufficient data to support this practice.

Barraclough et al. study is the largest study to date comparing ID with intramuscular (IM) HBV vaccination in hemodialysis patients nonresponsive to a primary HBV vaccination course. Study participants were reflective of dialysis patients in the developed world, making our results generalizable to this patient group. ID vaccination was well tolerated and convenient. They clearly show that ID vaccination afforded improved seroconversion, greater peak antibody titers, and similar duration of persistence of protective antibody titers. Although it is appropriate for IM HBV vaccination to remain the route of choice for primary vaccination in light of greater experience, proven efficacy, and ease of administration; our data suggest that ID vaccination provides a useful method for overcoming the immune deficit in the problematic group of patients nonresponsive to a primary vaccination course. A large randomized controlled trial is required to confirm their findings and the superiority of ID HBV vaccination in this setting [111].

## 13. Conclusion

It is very important to find more effective ways to prevent HBV infection in the society especially immunosuppressant and hemodialysis patients. However, infection rates are still unacceptably high, and further work is required. It is recommended that for ESRD patients double-dose vaccination be used at first as soon as possible probably before dialysis, and if it would not be effective; use adjuvanted vaccines for achieving better response, but there is a long way for finding better and more effective adjuvants in which have less side effects beside its protective properties. 
